# Searching for structure in collective systems

**DOI:** 10.1007/s12064-020-00311-9

**Published:** 2020-03-23

**Authors:** Colin R. Twomey, Andrew T. Hartnett, Matthew M. G. Sosna, Pawel Romanczuk

**Affiliations:** 1 Department of Biology, University of Pennsylvania, Philadelphia, PA USA; 2 West Hartford, CT USA; 3 Department of Ecology and Evolutionary Biology, Princeton University, Princeton, NJ USA; 4 Institute for Theoretical Biology, Department of Biology, Humboldt Universität zu Berlin, Berlin, Germany; 5 Bernstein Center for Computational Neuroscience, Berlin, Germany

**Keywords:** Collective behavior, Information theory, Coordination, Group structure

## Abstract

From fish schools and bird flocks to biofilms and neural networks, collective systems in nature are made up of many mutually influencing individuals that interact locally to produce large-scale coordinated behavior. Although coordination is central to what it means to behave collectively, measures of large-scale coordination in these systems are *ad hoc* and system specific. The lack of a common quantitative scale makes broad cross-system comparisons difficult. Here we identify a system-independent measure of coordination based on an information-theoretic measure of multivariate dependence and show it can be used in practice to give a new view of even classic, well-studied collective systems. Moreover, we use this measure to derive a novel method for finding the most coordinated components within a system and demonstrate how this can be used in practice to reveal intrasystem organizational structure.

## Introduction

In the absence of a quantitative definition, papers on collective behavior (including this one) often begin by listing well-known examples of collective systems, like fish schools or bird flocks. This gives a useful reference point for the reader, but offers little guidance on what to consider “collective” in other systems and behaviors. Even a canonical example of collective behavior like a fish school may vary in the degree of coordinated movement over time and transition between periods of ordered movement and disordered aggregation (Tunstrøm et al. 2013). Moreover, different parts of the same school may be more or less locally coordinated or preferentially coordinated with only subsets of the larger group (for example, in mixed species assemblies; Ward et al. 2018; Gil et al. 2018). Schools can also vary widely in size across and within species and environments. Millions of sardines moving together may be clearly collective, but a school of two is less clear.

Rather than searching for a heuristic distinction between “collective” and “not collective,” this paper investigates a principled measure of one of its defining characteristics: coordination. Typically coordination is measured in a system- and behavior-dependent way. For example, the average alignment of the headings of all the fish in a group provides a useful order parameter that indicates coordinated movement when high, and disordered aggregation when low (see, e.g., Couzin et al. 2002; Tunstrøm et al. 2013). While this same order parameter can be useful in other systems exhibiting collective movement, such as locusts (e.g., Buhl et al. 2006), it would have less utility for describing the degree of coordinated behavior in the nest-site selection process of honeybees (Seeley and Visscher 2004), bridge formation (Reid et al. 2015) and foraging decisions (Greene and Gordon 2007) in ants, social conflict policing in Macaques (Flack et al. 2006), quorum sensing in bacteria (Papenfort and Bassler 2016), or neuronal avalanches in slices of neocortex (Beggs and Plenz 2003). System-specific measures are useful in their relevant context, but make comparisons of coordination across systems or even between behaviors within the same system difficult to perform quantitatively.

Instead, in this paper we explore a system-independent measure of coordinated behavior based on a dimensionless information-theoretic measure of dependence. This measure quantifies the relative degree of statistical dependence shared by a set of elements (individuals) in any system, allowing the degree of macroscopic coordination to be quantified and compared across systems of any size. We demonstrate the practical utility of this measure in a classic model of collective behavior. Moreover, we provide a method that uses this measure to find the natural decompositions of a system into its most coordinated components. These decompositions provide mesoscale descriptions of the system that may offer a useful basis on which to make inferences about intermediate-scale social forces governing large-scale group behavior. Finally, we demonstrate the application of this method to both simulated and empirically recorded systems to show its utility in practice.

## Results

### Redundancy as a measure of coordination

Let $S = \{1, 2, \ldots , n\}$ be the indices of a set of random variables, $\{X_{i}\}_{i \in S}$, which in general may be neither identically distributed nor independent. In the context of a fish school or a bird flock, this could be the set of all the velocity vectors of the individuals in the group; for neurons, this could be the state of each neuron (firing or silent). In general, it could be any heterogeneous assemblage of the microscopic observables of a system. If we were asked to faithfully record the current state of the whole group, one strategy would be to simply write down a description of each element separately. One of the foundational results from information theory is that no lossless description of a random variable can be shorter on average than the tight lower bound given by its entropy (Shannon 1948). Thus a description of the system given by recording every element separately would require on average a minimum of $\sum _{i \in S} H(X_i)$ bits, where $H(X_i)$ is the entropy of $X_i$.

Alternatively, another strategy would be to instead write down a shared (or ‘joint’) description of all elements at once. A joint description can capitalize on the dependencies among a set of variables to reduce the overall description length needed. For example, to characterize the state of both a lamp and the light switch that controls it, one could simply record the on/off state of one of the two components. Knowing the state of either the switch or the lamp automatically tells us the state of the other, under perfect operating conditions. For less than perfect operating conditions, it will be necessary to include additional information about the state of the other component, but only as frequently as the light switch fails to determine the state of the lamp. In either case, the joint entropy of the lamp and the light switch together determines the lower bound on the lossless joint description of the system. Thus the smallest lossless joint description requires $H(\{X_i\}_{i \in S})$ bits on average, where we are guaranteed that $H(\{X_{i}\}_{i \in S}) \le \sum _{i \in S} H(X_i)$.

In fact, the only way in which the joint description is as costly as the sum of the individual (or ‘marginal’) descriptions is if all $X_i$’s are independent. The difference between the marginal and joint descriptions, given by

1 $$\begin{aligned} I(\{X_{i}\}_{i \in S})&= \sum _{i \in S} H(X_i) - H(\{X_{i}\}_{i \in S}), \end{aligned}$$

gives us a natural measure of how much we reduce the fundamental representation cost by using a joint, rather than a marginal, description. Another way to think about Eq. 1 is as a measure of redundancy: the amount of information that is made redundant (unnecessary) when describing $\{X_{i}\}_{i \in S}$ as a whole rather than by parts. A similar interpretation can be found in Watanabe (1960)’s original investigation of Eq. 1 as a general measure of multivariate correlation (also called “total correlation”).^1^

**Fig. 1:**
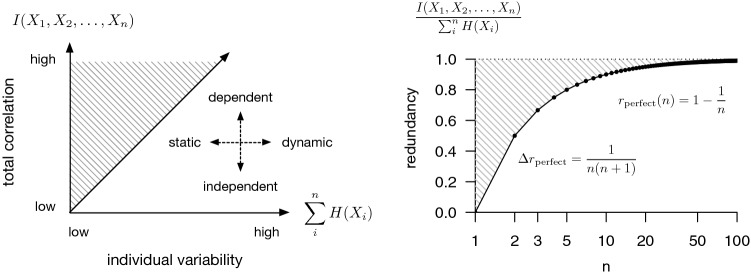
(*Left*) Schematic description of a system, $\{X_{i}\}_{i \in S}$, by its total correlation (y-axis), measuring dependence, and the total marginal entropy of its elements (x-axis). The 1–1 line and shaded area above are not achievable. Biological system examples may include starling flocks or fish schools (dynamic and dependent); whirligig beetle rafts (static and dependent); fish schools in a swarm (disordered) state (static and independent); and swarms of gnats (dynamic and independent). (*Right*) Feasible (white) and infeasible (shaded) redundancies for systems of a given size, *n*. The upper bound is given by a system in which every element is perfectly dependent on every other element (so knowing the state of one element is as good as knowing the state of every element in the system). The lower bound is zero, which occurs when all elements are independent

Notably, redundancy in the absolute sense given by Eq. 1 scales in magnitude with the size of the system. For example, if we take *n* identical copies^2^ of the same random variable, *X*, then we have $I(\{X_{i}\}_{i \in S}) = (n-1) H(X)$. This is a useful property for a measure of collective behavior, in the sense that just two or three of something behaving similarly is less “collective” than hundreds or thousands. On the other hand the $H(X)$ term indicates that this also scales with the magnitude of the individual variability in behavior (Fig. 1, *left*). This is orthogonal to what is typically meant by “collective.” A school of fish swimming slowly or quickly through the coral of a reef ought to be “collective” to the same degree provided their movement decisions depend on one another to the same degree, rather than depending additionally on the range and variability of individual decisions that could be made. To reflect this invariance to the magnitude of individual variability, it is useful to consider instead the relative redundancy (normalized total correlation), i.e.,

2 $$\begin{aligned} r = \frac{I(\{X_{i}\}_{i \in S})}{\sum _{i \in S} H(X_i)} = 1 - \frac{H(\{X_{i}\}_{i \in S})}{\sum _{i \in S} H(X_i)} = 1 - s, \end{aligned}$$

where *s* is then the proportion of non-redundant, or incompressible, information in the set. Using the same example as before, for *n* identical copies of *X*, $r = 1 - \frac{1}{n}$, which is invariant to $H(X)$, while still increasing with *n* (Fig. 1, *right*).

In general, the upper bound of relative redundancy for a fixed *n* is invariant to rescaling of the individual entropies, but sensitive to variability in the set of entropies. To see this, note that $H(\{X_{i}\}_{i \in S}) \ge \max _{i \in S} H(X_i)$, s.t.

3 $$\begin{aligned} 0 \le \frac{I(\{X_{i}\}_{i \in S})}{\sum _{i \in S} H(X_i)} \le 1 - \frac{\max _{i \in S} H(X_i)}{\sum _{i \in S} H(X_i)} < 1, \end{aligned}$$

for any set of $X_i$ (i.e., not necessarily all identical as in the prior example). Then rescaling all $H(X_i)$ by a constant factor does not change the upper bound, and the upper bound is closest to 1 when all $H(X_i)$ are equal. This last property also fits the intuitive definition of “collective,” in the sense that elements of a system behaving similarly should have similar variability in their individual behaviors.

To summarize, relative redundancy has the following properties useful for measuring coordination in collective behavior:

1. It increases the more the behavior of any one element in the system is informative about the behavior of all the other elements in the system.
2. Its upper bound increases as the number of individual elements in the system increases (yet remains on a zero to one scale).
3. It increases with increasing similarity in the variability of individual behavior.
4. It is invariant to the total amount of individual variability within the system.

As an example, swarms of gnats forming large mating groups would likely score low on this measure of collectivity (provided the microscopic property being measured is individual movement). While gnats within the swarm may have similar levels of variability in their velocities, their movements are relatively independent. In comparison, large groups of fireflies flashing in unison (provided the microscopic property measured is the on / off state of the firefly’s bioluminescent abdomen) should score high on the relative redundancy scale, regardless of species variability in the frequency of flashing. Relative redundancy should also give a graded distinction between “shoaling” and “schooling” in fish, based on the degree of coordinated movement behavior within the group (resulting in low and high relative redundancy, respectively).

#### Practical application

Computing relative redundancy in practice is challenging. Estimating the mutual information between just two variables (equivalently, the $n=2$ case for Eq. 1), or the entropy of a single variable, runs into sampling problems and issues of estimator bias (Paninski 2003). While there may be no universal solution, for systems with continuous microscopic properties (the quantities of each element of the system for which we would like to measure coordination across the system), we can still make progress by maximizing a lower bound on redundancy instead.

First, for continuous random variables that are marginally Gaussian with system-wide correlation matrix $P_S$, the Gaussian mutual information,

4 $$\begin{aligned} I_G(\{X_{i}\}_{i \in S}) = -\frac{1}{2} \log \det (P_S), \end{aligned}$$

is a lower bound on the total mutual information (Foster and Grassberger 2011; Kraskov et al. 2004). Since the marginals are continuous and Gaussian, each element has differential entropy

5 $$\begin{aligned} h_G(X_i) = \frac{1}{2} \log \left[ (2\pi e)^{k_i} \det (K_i) \right] , \end{aligned}$$

where $K_i$ is the covariance matrix of $X_i$, and $k_i$ is the number of variates of element *i*. Unfortunately, while $I_G(\cdot )$ is nonnegative, the differential entropy $h_G(\cdot )$ can be positive or negative. Fortunately, for an arbitrarily precise $\alpha$-bit *quantization* of $X_i$, its discrete entropy is approximated by $h(X_i) + \alpha$ (see Theorem 8.3.1 in Cover and Thomas 2006). Since the choice of $\alpha$ is arbitrary, we can choose it such that the differential entropies for the system are all positive. The choice of quantization cancels out in the numerator and only affects the denominator, giving

6 $$\begin{aligned} r&\ge \frac{I_G(\{X_{i}\}_{i \in S})}{\alpha + \sum _{i \in S} h_G(X_i)}, \end{aligned}$$

which is simple to compute in practice. However, since the quantization level, $\alpha$, changes the scaling, when making cross-system comparisons one must be sure to compute redundancy using the same $\alpha$ across all systems.

In general, when the random variables comprising the system are not marginally Gaussian, this lower bound can still be helpful. By substituting rank transformed variables $G_i$ for $X_i$ in the numerator, for which we enforce that each $G_i$ is marginally Gaussian distributed, the numerator remains a useful lower bound on the total correlation among the $X_i$ (by extension of Foster and Grassberger 2011; Kraskov et al. 2004, to the multivariate case). This essentially just measures the strength of any monotonic pairwise relationship among the system elements. The Gaussian differential entropies in the denominator are also upper bounds on the differential entropies of any continuous $X_i$ with the same means and (co)variances. Thus redundancy is lower bounded by these two quantities for any continuous $X_i$. Better or possibly even exact estimates of *r* may be possible depending on the system and microscopic variables at play; in any case, Eq. (2) still gives the correct system-independent blueprint for measuring coordination.

**Fig. 2:**
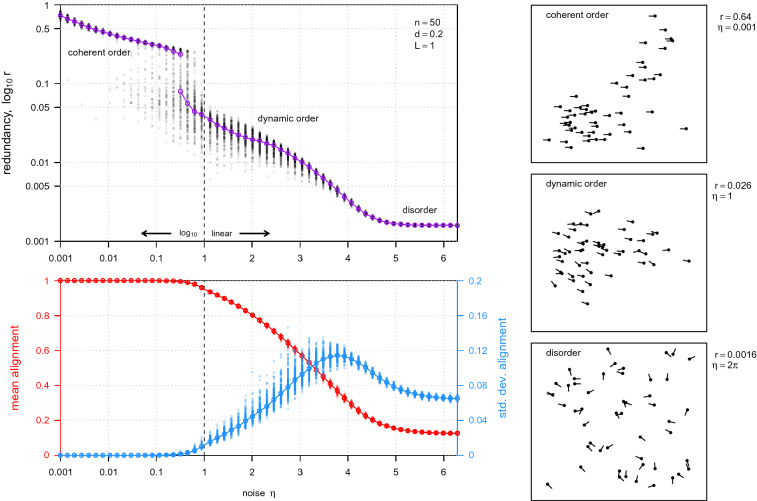
Example of redundancy as a measure of coordination in a system. (*Top left*) Redundancy for Vicsek model simulations (black dots) with $n = 50, d = 0.2, L=1$, and $\eta$ (noise in individual headings) varying along the x-axis. Simulations were run for 500 iterations to reach steady state, then redundancy was computed based on the subsequent 1000 iterations. Estimated mode(s) of simulation redundancy distributions at each noise level are shown (purple points); lines connect adjacent modes based on a threshold distance linking criterion. The x-axis scale is linear to the right of $\eta = 1$ (dashed vertical line), and $\log _{10}$ to the left. Qualitative descriptors of the system state, from disordered to dynamic order to coherent order are annotated on the plot. (*Bottom left*) Time-averaged mean alignment (red), the typical order parameter for the Vicsek model, as a function of noise, $\eta$. The standard deviation in mean alignment over time is also shown in blue (corresponding to blue axis at right). (*Right*) Snapshots of simulated systems in the coherent order (high redundancy), dynamic order (intermediate redundancy), and disordered (low redundancy) states. Agent positions (black points) and headings (black lines) shown in simulated two-dimensional space with periodic boundary conditions

As a simple numerical application using the above redundancy bound, Fig. 2 explores the Vicsek et al. (1995) model of collective motion with alignment only, i.e.,

7 $$\begin{aligned} \theta _i(t+1)&= \bar{\theta }_i(t) + \epsilon _i(t), \end{aligned}$$

8 $$\begin{aligned} \mathbf {x}_i(t+1)&= \mathbf {x}_i(t) + \mathbf {v}_i(t) \Delta t, \end{aligned}$$

where $\mathbf {x}_i(t)$ is the position of individual *i* at discrete time step *t*, $\mathbf {v}_i(t)$ is individual *i*’s velocity at time *t* given by its heading, $\theta _i(t)$, and a constant, *c* (fixed at 0.03 to match Vicsek et al. 1995), $\bar{\theta }_i(t)$ is the angular average heading of *i* and all neighbors within a distance *d* at time *t*, and $\mathbf {\epsilon }_i(t)$ is drawn i.i.d. from a uniform distribution on the interval $\left[ -\eta /2, \eta /2 \right]$. In this well-studied system, redundancy (Fig. 2, *Top left*) shows the same phase transition from disorder to order when varying the noise parameter $\eta$, as seen in the system-specific order parameter of average alignment (Fig. 2, *Bottom left*). Interestingly, it also shows an apparently discontinuous transition with a bistable region in the ordered regime, which to our knowledge has not been reported before. This appears to distinguish between “dynamic order” (in which there are still fluctuations in average alignment over time across the group) and “coherent order” (in which the group is almost always aligned). A detailed investigation of this transition is beyond the scope of this study and is left for future work. However, based on a visual inspection of the emergent dynamics, it seems likely that the observed discontinuous transition may be related to the correlation range of the orientation exceeding the finite system size, whereas the bistability emerges from different spatial configurations exhibiting either coherent or dynamic order for the same noise values.

### Redundancy partitioning for system structure

While relative redundancy (resp. incompressibility) can be used to compare the degree of collectivity exhibited by very different systems, it can also be used to characterize the dependency structure within a given system. Writing the relative redundancy as a function of a subset of the system, $A \subseteq S$, we have

9 $$\begin{aligned} r(A)&= 1 - \frac{H(\{X_{i}\}_{i \in A})}{\sum _{i \in A} H(X_i)}. \end{aligned}$$

What divisions of a system maximize the relative redundancy of each subset?

To make this question concrete, let $\widehat{S}$ be a set of indices for a collection of subsets of *S*, which we will refer to as the *components* of system *S*. That is, let $\widehat{S} = \{1, 2, \ldots , m\}$, where typically^3^$m \le n$, and introduce a probabilistic assignment *p*(*j*|*i*), $\forall (i,j) \in (S, \widehat{S})$,^4^ which can be read as the probability that element *i* belongs to component *j*. Then the expected quality of an assignment to a given component is

10 $$\begin{aligned} \mathbb {E}\left[ r(A)|j\right]&= \sum _{A \in \mathcal {P}(S)} r(A) p(A|j), \end{aligned}$$

where $\mathcal {P}(S)$ is the power set (set of all subsets) of *S*, and

11 $$\begin{aligned} p(A|j)&= \prod _{i \in A} p(j|i) \prod _{i \in {A}^{\mathsf {c}}} \left[ 1 - p(j|i)\right] , \end{aligned}$$

is the probability of subset *A* given the assignments of elements to component *j*, by a simple counting argument.^5^ Treating the quality of each component equally, the expected quality over all components is then

12 $$\begin{aligned} \mathbb {E}\left[ r(A)\right]&= \frac{1}{m}\sum _{j \in \widehat{S}} \mathbb {E}\left[ r(A)|j\right] . \end{aligned}$$

**Fig. 3:**
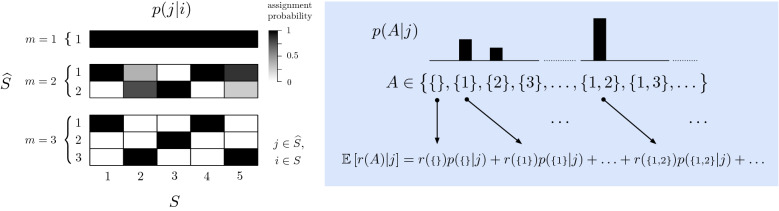
(*Left*) Example probabilistic assignments of $n = 5$ variables (i.e., $X_1, X_2, \ldots , X_5$), to $m = 1, 2, \text {and}\,3$ components. Probabilistic assignments of $i \in S$ to $j \in \widehat{S}$, written *p*(*j*|*i*), are shown as matrices of dimension *n* rows by *m* columns. Each cell is shaded according to the probability of assignment, ranging from 0 to 1 (white to black), with each column summing to 1. The $m = 2$ case illustrates a “soft assignment,” in which there are assignment probabilities between 0 and 1. The $m = 3$ case illustrates a “hard assignment,” in which each assignment probability is only either 0 or 1. (*Right*) The “quality” of the *j*-th component is measured in terms of its expected redundancy: $\mathbb {E}\left[ r(A) | j \right]$. The expectation is over the distribution of possible sets, *p*(*A*|*j*), which is a function of the probabilistic assignments *p*(*j*|*i*) (see Eq. 11)

Note that the redundancy of any individual element, i.e., $r\left( \{1\}\right)$, is equal to zero according to Eq. 9. For continuity, we define the redundancy of the empty set, $r\left( \{\}\right)$, to be zero. A visual example of dividing a system into different numbers of components and measuring component redundancy is illustrated in Fig. 3.

#### Rate-distortion theory

While this gives us a natural way to evaluate the quality of a given assignment, it does not immediately provide us with a way to find such an assignment. Instead, we draw inspiration from the information-theoretic treatment of compression given by rate-distortion theory (see Shannon 1959; Cover and Thomas 2006). Classical rate-distortion theory addresses the following problem: given a source (random variable) *X*, a measure of distortion, *d*, and an allowable level of average distortion *D*, determine the minimum amount of information necessary for a compressed description of *X* that introduces an average distortion no more than *D*. I.e.,

13 $$\begin{aligned} R(D) = \min _{p(\hat{x}|x)\,:\,\mathbb {E}d(x,\hat{x})\,\le \,D} I(X;\widehat{X}), \end{aligned}$$

where the rate, *R*(*D*), equals the minimum amount of information (measured in bits per symbol, hence “rate”) needed for average distortion *D*. In this case, the rate measures the information, $I(X;\widehat{X})$, that the compressed representation, $\widehat{X}$, needs to keep about the source, *X*, where

14 $$\begin{aligned} I(X;\widehat{X})&= \sum _{x,\hat{x}} p(x,\hat{x}) \log \frac{p(x,\hat{x})}{p(x)p(\hat{x})} \end{aligned}$$

is the mutual information between *X* and $\widehat{X}$. The lower the rate, the better the compression, but (depending on the source and the distortion measure) the higher the average distortion introduced. Surprisingly, not only can the rate-distortion curve be characterized numerically in general, the minimal compressed representation of *X* can be found via a simple, iterative, alternating minimization algorithm (Blahut 1972; Arimoto 1972).

#### Redundancy partitioning

Though there are important differences from rate-distortion theory (discussed in “Appendix 1”), we can similarly frame the problem of finding structure based on redundancy as a compression problem. Here, we wish to find the assignment of elements of *S* to components of $\widehat{S}$ that achieves an average redundancy no less than $r^*$, and otherwise preserves as little about the original identities of the elements as possible. I.e.,

15 $$\begin{aligned} R(r^*) = \min _{p(j|i)\,:\,\mathbb {E}\left[ r(A)\right] \,\ge \,r^*} I(S;\widehat{S}), \end{aligned}$$

where *p*(*j*|*i*) is further required to be nonnegative and sum to one. This is not a standard rate-distortion problem, but we can use many of the same ideas developed by Blahut (1972) and Arimoto (1972) in their original numerical algorithms for deriving a practical solution. We give a brief account of this derivation here; see “Appendix 1” for a complete account.

Introducing Lagrange multipliers to constrain the $\sum _{j \in \widehat{S}} p(j|i) = 1$ (non-negativity will be enforced by the form of the solution), the variational problem becomes

16 $$\begin{aligned} L\left[ p(j|i) \right]&= I(S;\widehat{S}) - \beta \sum _{j \in \widehat{S}, A \in \mathcal {P}(S)} r(A) p(A|j) + \sum _{i \in S} \lambda (i) \sum _{i \in \widehat{S}} p(j|i), \end{aligned}$$

where $\beta$, the Lagrange multiplier for the average redundancy constraint, absorbs the 1/*m* term. Taking the derivative with respect to a particular $j'$ and $i'$, we have

17 $$\begin{aligned} \frac{\partial }{\partial p(j'|i')} L\left[ p(j|i) \right]&= p(i') \log \frac{p(j'|i')}{p(j')} - \beta \sum _{j \in \widehat{S}, A \in \mathcal {P}(S)} r(A) \frac{\partial p(A|j)}{\partial p(j'|i')} + \lambda (i'), \end{aligned}$$

where

18 $$\begin{aligned} \frac{\partial p(A|j)}{\partial p(j'|i')}&= \left\{ \begin{array}{ll} 0 &{}\quad \text{ if } \,\, j \ne j', \\ f_{i'}(A|j') &{}\quad \text{ if } \,\, j = j', i' \in A, \\ -f_{i'}(A|j') &{}\quad \text{ if } \,\, j = j', i' \in {A}^{\mathsf {c}}, \end{array} \right. \end{aligned}$$

and

19 $$\begin{aligned} f_i(A|j)&= \prod _{k \in A \setminus \{i\}} p(j|k) \prod _{k \in {A}^{\mathsf {c}} \setminus \{i\}} \big [1 - p(j|i)\big ], \end{aligned}$$

where $A \setminus \{i\}$ is the relative complement of the singleton set $\{i\}$ with respect to *A*.

Then setting $\partial L / \partial p(j'|i') = 0$ and splitting the sum over $\mathcal {P}(S)$ into terms with and without $i' \in A$, we have

20 $$\begin{aligned} \begin{aligned} p(i') \log \frac{p(j'|i')}{p(j')}&= \beta \sum _{\{A \in \mathcal {P}(S)\,:\,i' \in A \}} r(A) f_{i'}(A|j') \\&\quad - \beta \sum _{\{A \in \mathcal {P}(S)\,:\,i' \in {A}^{\mathsf {c}} \}} r(A) f_{i'}(A|j') \\&\quad - \lambda (i'). \end{aligned} \end{aligned}$$

Let

21 $$\begin{aligned} d(i,j)&= \frac{1}{p(i)} \sum _{\{A \in \mathcal {P}(S)\,:\,i \in A \}} r(A) f_{i}(A|j), \end{aligned}$$

and define $d_{\mathsf {c}}(i,j)$ to be identical except substituting $i \in {A}^{\mathsf {c}}$ for $i \in A$. Lastly, let $\Delta d(i, j) = d(i,j) - d_{\mathsf {c}}(i,j)$. Then, dividing through by $p(i')$ and substituting, we have,

22 $$\begin{aligned} \log \frac{p(j'|i')}{p(j')}&= \beta \Delta d(i',j') - \frac{\lambda (i')}{p(i')}. \end{aligned}$$

Finally, substituting $\log \mu (i') = \lambda (i') / p(i')$ and solving for $p(j'|i')$,

23 $$\begin{aligned} p(j'|i')&= \frac{p(j')}{\mu (i')} e^{\beta \Delta d(i',j')}. \end{aligned}$$

Enforcing the constraint that $\sum _{j \in \widehat{S}} p(j|i') = 1$ and simplifying notation, we have

24 $$\begin{aligned} p(j|i)&= \frac{p(j) e^{\beta \Delta d(i,j)}}{\sum _{j' \in \widehat{S}} p(j') e^{\beta \Delta d(i,j')}}. \end{aligned}$$

Before moving on, it is worth noting that $\Delta d(i,j)$ has a simple and intuitive interpretation. It is the difference in redundancy for component *j* when *i* is included versus when it is excluded, weighted by the relative importance of *i*.

Note that *p*(*j*) and *p*(*A*|*j*) depend on the choice of *p*(*j*|*i*). The final algorithm,

25 $$\begin{aligned} \left\{ \begin{array}{rl} p_t(j|i) &{}= \frac{p_t(j) e^{\beta \Delta d(i,j)}}{\sum _{j' \in \widehat{S}} p_t(j') e^{\beta \Delta d(i,j')}}, \\ p_{t+1}(j) &{}= \sum _{i \in S} p_t(j|i) p(i), \\ p_{t+1}(A|j) &{}= \prod _{i \in A} p_t(j|i) \prod _{i \in {A}^{\mathsf {c}}} \big [1 - p_t(j|i) \big ],\\ \end{array} \right. \end{aligned}$$

follows a similar alternating minimization scheme to the one developed by Blahut and Arimoto and generalized by Csiszár and Tsunády (1984), albeit with only local optimality guarantees similar to Tishby et al. (1999); Banerjee et al. (2005). See "Appendix 1" and Fig. 8 for a complete derivation and description of the algorithm.

One immediate issue is the $2^n$ scaling of the number of subsets of *S* as *n* (the number of elements of *S*) increases. First, it is worth noting that there are non-trivial collective systems of empirical interest even for small *n*. Current computational hardware may permit exact computation up to around $n \approx 15$ even on consumer hardware, which would be relevant for many experimental systems (as in, e.g., Miller and Gerlai 2007; Katz et al. 2011; Jolles et al. 2018). Second, for larger systems, Monte Carlo estimation of $\Delta d(i,j)$ can be readily employed, e.g., for *K* samples,

26 $$\begin{aligned} \begin{array}{rll} \widehat{d}(i,j) &{}= \displaystyle \frac{1}{p(i) K} \sum _{k = 1}^K r\big (A_{ij} \cup \{i\}\big ),\\ \widehat{d}_{\mathsf {c}}(i,j) &{}= \displaystyle \frac{1}{p(i) K} \sum _{k = 1}^K r\big (A_{ij} \setminus \{i\}\big ), &{}\quad \text {where}\,A_{ij} \sim f_i(\cdot |j). \end{array} \end{aligned}$$

For large systems in particular initializing near good solutions may be helpful. In many systems we may expect elements to be spatially or temporally dependent, and use that prior knowledge to initialize reasonable clusters. However the preliminary results given in the next section do not employ any such strategy; we simply run the algorithm many times beginning with many different initial conditions and select the best solution generated. Finally, although we omit the exposition here, in the “hard-partition” limit (as $\beta \rightarrow \infty$), *p*(*j*|*i*) becomes a delta function, meaning that no sampling is necessary and we need only consider adding or dropping each element from each component on each iteration. When using the Gaussian bound on redundancy introduced in "Practical application" section, this can be accomplished in $O(n^4)$ (or $O(n^3)$ with some decrease in numerical precision). Our open source implementation of this algorithm is available by request or online at https://github.com/crtwomey/sscs.

### Experiments

#### Simulation experiments

We tested the proposed algorithm on two sets of data: simulations of schooling groups, and empirical data collected from the movements of schooling fish in a lab environment. The former allow us to control the dependency structure of the system, while the latter allows us to demonstrate applicability to empirical systems. Simulations used a simple model of coordinated movement based on attraction, alignment, and repulsion social forces (based on Romanczuk et al. 2012; Romanczuk and Schimansky-Geier 2012; a description of the model and additional information on the simulation conditions can be found in Appendix 2). Position and velocity data for independent groups of size $n = 5,\,10,$ and 20 were generated for a high $(\eta = 0.2)$ and low $(\eta = 0.15)$ noise conditions.

#### Empirical experiments

Movement data of fish comes from videos originally recorded by Katz et al. (2011). In that work, groups of 10, 30, and 70 golden shiners (*Notemigonus crysoleucas*) were purchased from Anderson Farms (www.andersonminnows.com) and filmed in a $1.2 \times 2.1\,\mathrm {m}$ tank with an overhead camera. Videos were then corrected for lens distortion and fish were tracked using the same custom in-house software developed by Haishan Wu and used in Rosenthal et al. (2015). The software begins by detecting all individuals in each frame, then links individuals across frames to form tracks. All tracks were manually corrected to ensure accuracy. Individual positions and velocities were estimated from these tracks using a $3^\mathrm{rd}$ order Savitzky–Golay filter (Savitzky and Golay 1964; similar to, e.g., Harpaz et al. 2017) with a 7 frame smoothing window (videos were recorded at 30 fps). Interactions between fish are time-dependent; for the results presented here we simply chose a fixed window of $\pm \, 15\,\mathrm {s}$ surrounding a given time *t* to estimate the dependency structure of the group. An optimal choice of time window is left for future work.

#### Experimental results

**Fig. 4:**
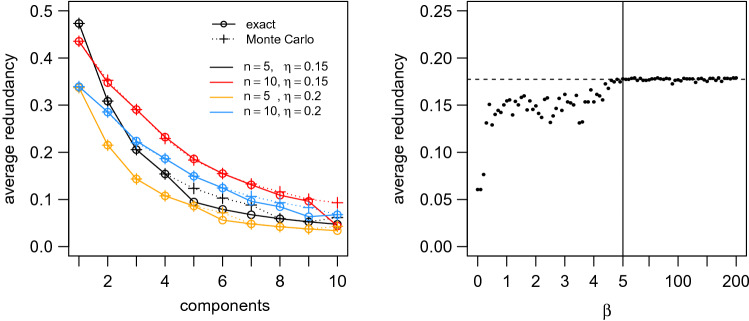
Algorithm implementation and parameter sensitivity. (*Left*) Comparison of exact and Monte Carlo estimates of $\Delta d(i,j)$, for groups of size 5 and 10, for low and high noise conditions. Note that the number of components can be greater than the number of elements; in this case some components will be empty, i.e., have no elements assigned to them. (*Right*) Impact of the choice of $\beta$ on the average redundancy of the recovered components for a simulated group of size 10, high noise condition, searching for 5 components. Dotted line shows the mean of the solutions for $\beta > 5$

The algorithm outlined in "Redundancy partitioning" section requires specifying the number of components and a parameter, $\beta$, which controls the relative importance of maximizing the average redundancy of the components as opposed to maximally compressing the original set of system elements. While it will be interesting to investigate the ‘soft-partitioning’ aspect of this approach in future work, here we simply consider the hard assignment case, which requires only that $\beta$ is large. Figure 4 (*Right*) illustrates this point, showing the stabilization of average component redundancy for $\beta > 5$. We found that $\beta = 200$ was sufficient to recover hard assignments in all cases tested here.^6^ Since relative redundancy ranges between 0 and 1 for any dataset, these parameter values should generalize well to other systems, and leaves the method free of parameter fine-tuning.

To validate that the Monte Carlo estimate of $\Delta d(i,j)$ employed is effective, we compared its behavior to exact computations of $\Delta d(i,j)$ for small system sizes (simulated groups of size 5 and 10). We ran each version of the algorithm for up to 10 components and took the best (maximum) average component redundancy achieved over 100 random initializations of the assignment matrix *p*(*j*|*i*). Figure 4 (*Left*) shows that the results are in good agreement, and where there are discrepancies they tend to favor the Monte Carlo method, in that the Monte Carlo method recovers solutions with higher average redundancy.

**Fig. 5:**
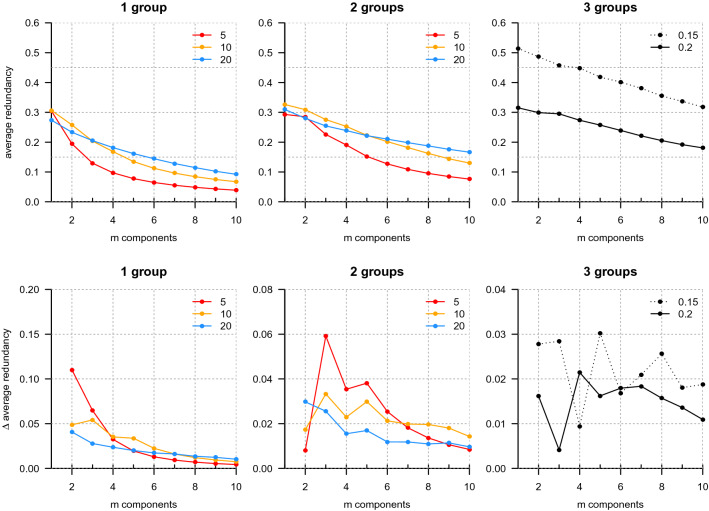
Partitioning results for simulations of 1, 2, and 3 independent (non-interacting) groups. Top row shows average redundancy as a function of the number of components (*m*). Bottom row shows the magnitude of the change in average redundancy between *m* and $m-1$ components (larger values are larger decreases). (*Left*) For single cohesive groups of size $n =$ 5, 10, or 20, the average redundancy (y-axis) of the components decreases approximately monotonically as the number of components increases. The $n = 10$ group has shallow local minima in $\Delta$ average redundancy at $m = 2$ and 4. (*Center*) For two non-interacting groups of the same size, the average redundancy approximately plateaus at two components for $n = 5$ and 10. For $n = 20$, the first minimum in $\Delta$ average redundancy is achieved at $m = 4$. (*Right*) A mixed (varying in group size) collection of three non-interacting groups, with sizes 5, 10, and 20, first plateau in average redundancy at three or four components, depending on the noise $(\eta )$ used in the simulation. For comparison, the left two plots show results for $\eta = 0.2$ (the ‘high’ noise)

Next, we tested the algorithm on simulated data in which the dependency structure of the simulated groups was known, using the hard partitioning variant of the algorithm for computational efficiency. For each test, we computed the maximum average component redundancy recovered for up to 10 components, again using 100 random initializations of the assignment matrix for each computation. In all cases partitioning decreases the average redundancy of the system with increasing number of components (Fig. 5).^7^ However the magnitude of the change in average redundancy (or ‘$\Delta$ average redundancy’) from *m* to $m-1$ components is informative of the system’s dependency structure. Small values of $\Delta$ average redundancy occur when subdividing the system has a comparatively minor impact on average redundancy, which should be expected when partitioning relatively independent parts of the system. In comparison, a large increase in the value of $\Delta$ average redundancy appears to occur when a strongly interacting component is split. This can be seen by comparing the $\Delta$ average redundancy curves for each group size between instances of a single group (Fig. 5*Left*) in the system or two independent, non-interacting groups in the same system (Fig. 5*Middle*). The $\Delta$ average component redundancies for systems containing only a single group have either no or only shallow local minima followed by at most small increases. In comparison, $\Delta$ average redundancies for systems with two non-interacting groups, in pairs of matched size groups of 5, 10, and 20, have comparatively deep local minima first occurring at 2 components for $n =$ 5 and 10, and at 4 components for $n = 20$, followed directly by relatively large increases in Δ average redundancy. At the point preceding each of these transitions from low to high $\Delta$ average redundancy, the two non-interacting groups are assigned to separate components by the algorithm, and in the $n = 20$ case the two groups are further subdivided into two spatially assorted components each. Finally, the $\Delta$ average redundancies for a system of three non-interacting groups of mixed sizes 5, 10, and 20 were computed, with local minima first occurring at 3 and 4 components for high and low noise conditions, respectively (Fig. 5*Right*), followed by large increases in $\Delta$ average redundancy.^8^ Taken together, this is evidence that the transition from low to high $\Delta$ average component redundancies recovered by the algorithm reflect the dependency structure of the underlying system. It suggests that these features may be useful in identifying relevant structure in other systems, even those with less extreme dependency structures.

**Fig. 6:**
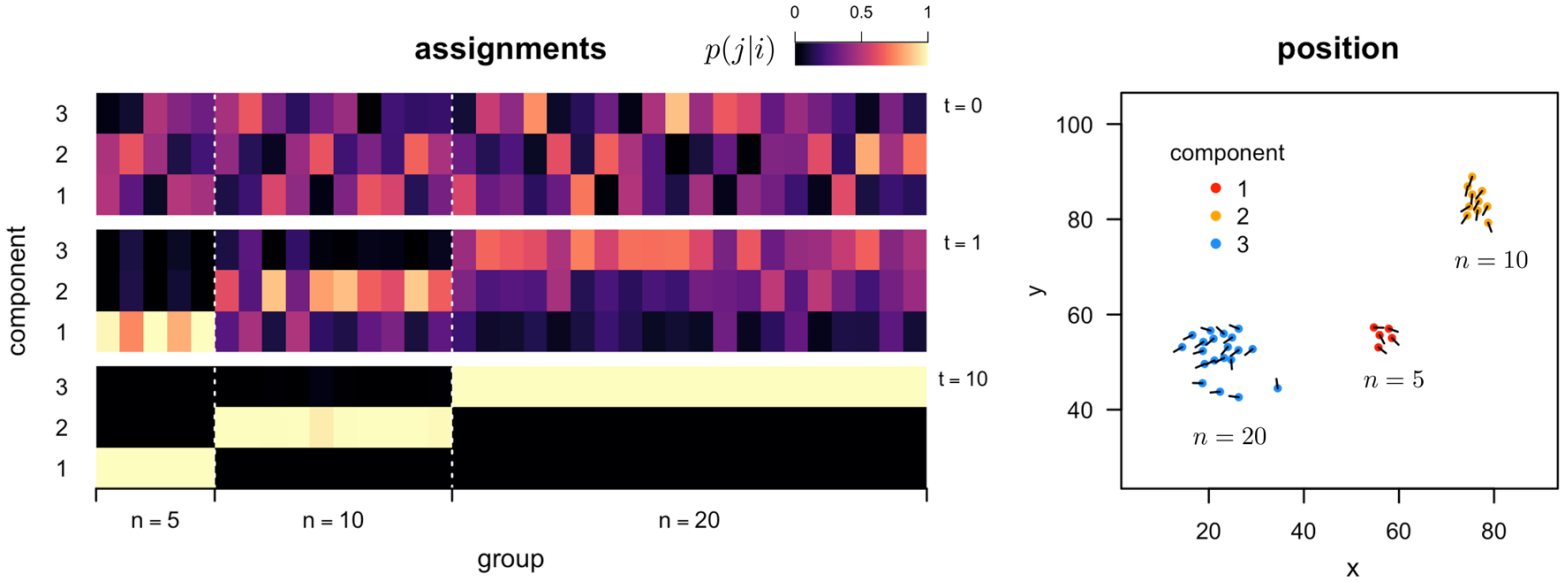
Generation of assignments by the average redundancy partitioning algorithm for a mixture of three non-interacting simulated groups, based on coordination in individual velocities. (*Left*) Assignments generated by the proposed sequential algorithm for three components after initialization $(t = 0)$, 1 iteration $(t = 1)$, and 10 iterations $(t = 10)$, at *top*, *middle*, and *bottom*, respectively. The color scale indicates the probability of assigning a member of a group (column) to a particular component (row), where low to high probability is coded dark to light (color scale top right). Original groupings of the system into its three non-interacting subsets are indicated on the x-axis. (*Right*) Two-dimensional positions (arbitrary units) of simulated system at one time point, color-coded by final component assignment; velocity vectors indicated by line segments. The algorithm correctly separates each subgroup based on coordination in velocities alone, without reference to spatial position

Figure 6 illustrates the iterative generation of assignments for the algorithm in the mixed three group (high noise) case. Assignments change and harden until they converge on a (local) maximal average redundancy partition of the system’s elements (*Left*). The assignments generated by the algorithm of system elements to components correspond one-to-one with the original, non-interacting set of three groups (of sizes 5, 10, and 20) comprising the whole system (of total size 35). Positions of the elements of the system and their velocity vectors are shown for one time point, colored by the component they were assigned to (which corresponds to their original group), in Fig. 6 (*Left*). Note that, while the snapshot shown in Fig. 6 was chosen to show the three distinct groups, at many points in the simulation the positions, velocities, or both, overlapped between the three groups. The algorithm is able to recover the independent groups in the system without using spatial position information, based on coordination in individual velocities alone.

**Fig. 7:**
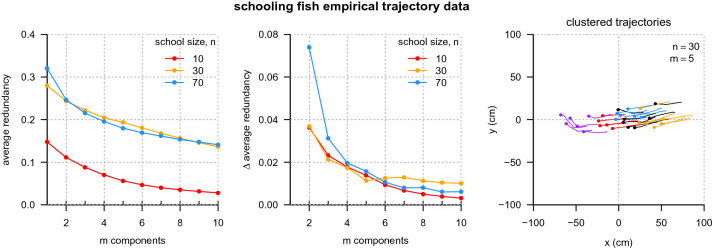
Coordinated substructure for empirical fish schools. (*Left*) Average component redundancy as a function of the number of components, for fish groups of size $n = 10$, 30, and 70. (*Middle*) Magnitude of the change in average component redundancy between *m* and $m-1$ components. The $n = 30$ school (orange lines) has a local minimum at $m = 5$ components. (*Right*) Example partitioning of a group of size 30 fish into five components (shown as different colors). Dots indicate the positions of the fish (swimming freely in a large $1.2\,\mathrm {m} \times 2.1\,\mathrm {m}$ arena) relative to the school mean position. Line segments indicate historical positions of each individual 2 s into the past

**Fig. 8:**
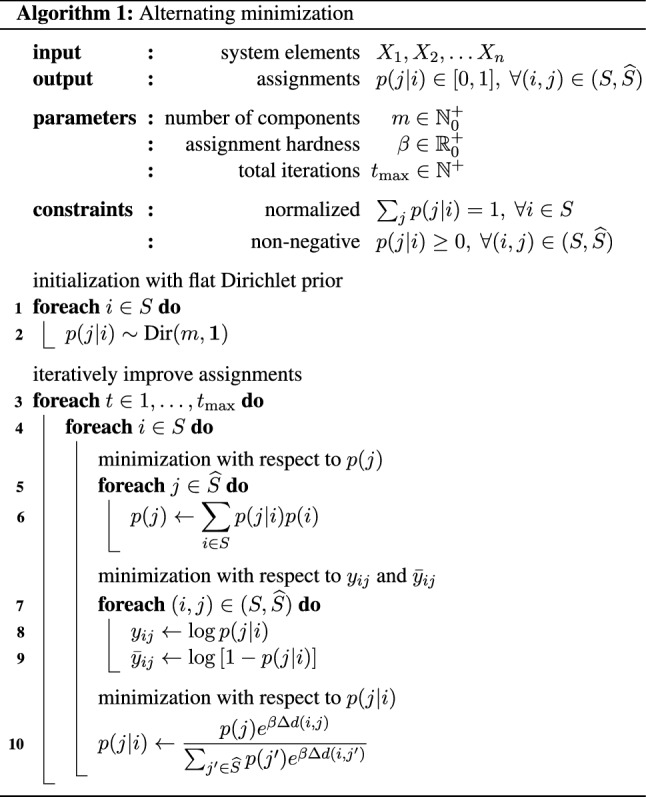
$\mathbb {N}^{+}$ are the positive integers, while $\mathbb {N}^{+}_0$, $\mathbb {R}^{+}_0$, are the nonnegative (positive including zero) integers and real numbers, respectively. For hard clustering, $\beta$ just needs to be large. Parameter $t_{\max }$ needs to be large enough for convergence; alternatively, it can be replaced by a criterion based on a minimum difference in improvement between iterations. Lines 2 and 10 are to be understood as vector operations over the set $j \in \widehat{S}$

Finally, we applied the algorithm to empirical data collected on fish schools to validate that the method is able to recover sensible results for strongly interacting groups and from non-simulated data. Figure 7 shows that for fish, groups of size 10 interact strongly enough (in at least the one instance tested here) to be considered one coherent unit, while groups of size 30 are already large enough to have subsets that more strongly interact with one another than the rest of the group (e.g., the local minima in $\Delta$ average redundancy at $m = 5$ components; Fig. 7*Middle*). The component assignments at the $m = 5$ local minima and positions for the school of 30 fish are shown in Fig. 7 (*Right*) at a single time point. The subdivisions of the system show strong spatial assortment with a stratification of the group from front to back. As in the simulation case, here we use only coordination in individual velocities to determine partitions, so this spatial assortment is a consequence of similar behavior as opposed to some criterion based on proximity. Further work is needed to investigate the duration of substructure in fish schools, as well as the emergence and disappearance of components over time.

## Discussion

**Redundancy as a measure of coordination**

Collective behavior is an emergent property of the actions and interactions of a system’s constituents. One of its characteristic features is a high degree of coordination among the individual elements of a system. In this work, we explored an information-theoretic measure of coordination defined by relative redundancy, or one minus the ratio of the maximally compressed description of a system to its uncompressed description. This quantity can be compared between systems of any size and total variability, and in this sense it functions as a system-independent measure of coordination. As a numerical example, we showed that measuring redundancy for a simple model of collective motion exhibited both the classic transition from order to disorder in this system as the noise in individual headings increases, but interestingly, also identified an apparently discontinuous transition from “coherent order” to “dynamic order.” While these results should be investigated more systematically in future work, they demonstrate the practical utility of this measure and suggest it may be used to shed new light on even classic models of collective behavior.

Still, redundancy as a universal measure of coordination is challenging to compute in general. While the Gaussian bound introduced in "Practical application" section is useful in practice, it has limited applicability. It is not appropriate for discrete systems or mixed measurements of continuous and discrete individual properties. One potentially promising direction for future work is to better constrain empirical estimates of redundancy by leveraging the rapidly growing body of research devoted to inferring and modeling individual-level interaction rules in a wide range of natural systems (see, e.g., Ballerini et al. 2008; Lukeman et al. 2010; Nagy et al. 2010; Katz et al. 2011; Herbert-Read et al. 2011; Bialek et al. 2012; Strandburg-Peshkin et al. 2013; Rosenthal et al. 2015; Harpaz et al. 2017; Torney et al. 2018; Hein et al. 2018; Sosna et al. 2019). Accurate probabilistic models of individual behavior can be used to estimate the time-varying relative redundancy of empirically recorded configurations of individuals. This could be useful for improving system-specific estimators of redundancy, which may be of particular importance when making cross-species comparisons; for example, to understand the evolution of collective behavior.

Finally, while coordinated behavior is central to what it means to be collective, it is not the only important property of collective systems. In the Vicsek model of collective motion explored in Fig. 2, coordination is highest in the “coherent order” state, in particular when the entire system is locked into a single stable heading. Collective behavior in living systems can rarely afford to be so rigid; animal groups must respond and adapt appropriately to their environment. Daniels et al. (2016)’s investigation of amplification, i.e., the extent to which individuals within the group can affect group-level properties, is of particular interest in this regard.

**Redundancy partitioning for system structure**

There are a wide range of both general purpose clustering algorithms (see Jain 2010; Xu and Tian 2015) and network community detection methods (see Forunato 2010), owing to a diversity of plausible clustering and community detection criteria. The justification for the average relative redundancy criterion presented here stems from its principled approach to the specific problem of quantifying coordination and its demonstrated ability to identify dependent structure in collective systems. It is specific in scope and not intended as a drop-in replacement for other clustering methods for arbitrary similarity matrices.

This approach to understanding the structure of collective systems also differs from methods concerned with the inference of individual interaction networks. For one, this method makes no attempt to construct such a network. Useful information-theoretic methods based on, e.g., estimating the transfer entropy (Lizier and Rubinov 2012) or causation entropy (Lord et al. 2016) between and among system elements can be used for this purpose. Similarly, when individuals in a group need to each remember their own representation of within-group interactions, as in Macaques, biologically plausible interaction representations can be inferred based on a sparse coding principle (Daniels et al. 2012). Instead, this approach attempts to simply identify the maximally coordinated components of a system, which offers a natural mesoscopic locus of analysis for the full system’s behavior. It could then be interesting to study the network of transfer or causation entropy between coordinated components, for instance, though this is made potentially more challenging by the possibly only short-term persistence of any given component.

There are many questions left for future work. First, the identification of transitions from low to high $\Delta$ average redundancy with increasing number of components is only a heuristic. In some cases there may be no local minima, or there may be multiple, in which case there may be more than one useful decomposition of the group. In other cases it may be more appropriate to divide the group into a given number of components regardless of the existence or position of a minimum. Further theoretical work is needed on the significance of plateaus in the average redundancy plot; we present only empirical evidence of their utility here. Second, an investigation of these features as a function of the time window chosen for computing the dependency structure may be important for understanding how the dependency structure of the group scales with time. It might be expected that on short time-scales for many systems only very local interactions will matter, requiring many components, while on longer time scales the system may be best represented as a single component.

It may also be important to investigate the algorithm presented here in the context of generating a soft-partitioning of a system’s elements into partially overlapping components. Using intermediate values of $\beta$ may allow the algorithm to find better average redundancy solutions ‘in-between’ *m* and $m+1$ components, in which assignments may be shared among components. At the same time, since optimal sets of components are not guaranteed to be unique, it may be important to explore the set of equally (or nearly equally) optimal solutions as an ensemble of equivalent descriptions of a system. Moreover, exploring the range of solutions as the number of components varies may reveal whether or not the system exhibits some form of hierarchical structure. In hierarchical systems we would expect components to be successively subdivided as the number of components increases.

One practical application of this method could be to the principled identification of a “group” in fission-fusion systems where this is an amorphous, time-varying concept. Another potential application of the method may be to long time-series, where the dependency structure itself is dynamic. Characterizing the natural decompositions of a system as a function of time may reveal important time-dependent mesoscopic features. How does the natural number of components of a system fluctuate in time, and how long do components persist? How do they interact as a function of time? These questions are central to the study of collective systems and may benefit from the quantitative approach to measuring coordination and identifying group structure introduced here.

## Figures and Tables

**Fig. 9:**
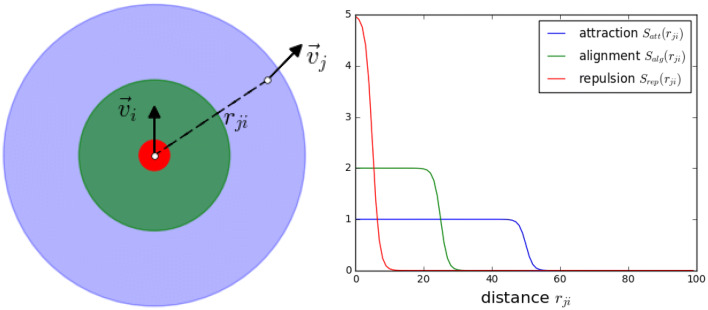
(*Left*) Schematic of the effective social interactions, with repulsion dominating at short distances (red zone), attraction dominating at large distances (green zone) and main contribution of alignment at intermediate ranges (blue zone). (*Right*) The strength of the different social forces versus distance for the different interactions

## Data Availability

Empirical video data for schooling fish provided by Iain D. Couzin, from the work of Katz et al. (2011). I.D.C. contributed directly to the filming and collection of this data. Special thanks also to Joshi Leibrock for additional simulations and insight into the $\Delta$ average redundancy heuristic.

## 1 Algorithm

Here we give an expanded account of the redundancy compression algorithm.

### 1.1 Rate-distortion compression

Classical rate-distortion theory treats the following optimization problem:

27 $$\begin{aligned} \begin{array}{lcll} \displaystyle \underset{p(\hat{x}|x)}{\text {minimize}} \quad &{} {I(X;\widehat{X})} &{}&{}\\ \text {subject to} \quad &{} \mathbb {E}[d(x,\hat{x})] &{}\le D &{} \\ &{} p(\hat{x}|x) &{}\ge 0 &{} \quad \forall (x,\hat{x}) \in (X,\widehat{X}) \\ &{} \sum _j p(\hat{x}|x) &{}= 1 &{} \quad \forall x \in X, \end{array} \end{aligned}$$

where

28 $$\begin{aligned} \mathbb {E}\left[ d(x,\hat{x})\right]&= \sum _{\hat{x} \in \widehat{X}} \sum _{x \in X} p(\hat{x}|x)p(x)d(x,\hat{x}), \end{aligned}$$

and *p*(*x*) is given. The problem as stated is not convex due to the form of $I(X;\hat{X})$. However, writing the objective as

29 $$\begin{aligned} I(X;\widehat{X})&= \sum _{x,\hat{x}} p(\hat{x}|x)p(x) \log p(\hat{x}|x) - \sum _{x,\hat{x}} p(\hat{x}|x)p(x) \log p(\hat{x}), \end{aligned}$$

it is clear that the problem is convex when varying $p(\hat{x}|x)$ or $p(\hat{x})$ separately, holding the other constant. Since the distortion constraint, $\mathbb {E}\left[ d(x,\hat{x})\right]$ is convex in $p(\hat{x}|x)$, the problem can be restated as a convex double minimization of the form

30 $$\begin{aligned} \min _{p(\hat{x}|x)} \min _{p(\hat{x})} I(X;\widehat{X}), \end{aligned}$$

which is minimized for fixed $p(\hat{x}|x)$ by

31 $$\begin{aligned} p(\hat{x}) = \sum _x p(\hat{x}|x) p(x), \end{aligned}$$

and for fixed $p(\hat{x})$ by

32 $$\begin{aligned} p(\hat{x}|x)&= \frac{p(\hat{x})\exp \left[ -\beta d(x,\hat{x}) \right] }{\sum _{\hat{x}'} p(\hat{x}') \exp \left[ -\beta d(x,\hat{x}')\right] }, \end{aligned}$$

(see Blahut 1972; Arimoto 1972; Cover and Thomas 2006). This leads to the classic Blahut-Arimoto algorithm, which, by iterative application of these two self-consistent equations for a given $\beta$, converges to an optimal solution point on the rate-distortion curve with tangent slope equal to $\beta$.

### 1.2 Redundancy compression

In this paper, we are interested in a similar problem:

33 $$\begin{aligned} \begin{array}{lclll} \displaystyle \underset{p(j|i)}{\text {minimize}} &{}{I(S;\widehat{S})} &{}&{}&{}\\ \text {subject to} &{} \mathbb {E}[r(A,j)] &{} \ge &{} r^* &{} \quad \forall j \in \widehat{S} \\ &{} p(j|i) &{} \ge &{} 0 &{} \quad \forall (i,j) \in (S,\widehat{S}) \\ &{} \sum _j p(j|i) &{} = &{} 1 &{} \quad \forall i \in S, \end{array} \end{aligned}$$

where

34 $$\begin{aligned} \mathbb {E}\left[ r(A,j)\right]&= \frac{1}{m} \sum _{j \in \widehat{S}} r(A,j) \end{aligned}$$

and

35 $$\begin{aligned} r(A,j)&= \sum _{A \in \mathcal {P}(S)} r_A \prod _{i \in A} p(j|i) \prod _{i \in {A}^{\mathsf {c}}} \left[ 1-p(j|i)\right] . \end{aligned}$$

The fixed 1/*m* weighting of the marginal importance of each component, *j*, in the redundancy constraint, $\mathbb {E}\left[ r(A,j)\right]$, is a minor variation from the classical rate-distortion problem. The important difference is that the *r*(*A*, *j*) inequality constraint is not convex with respect to *p*(*j*|*i*). However, with change of variables $b_A = \log r_A$, $y_{ij} = \log p(j|i)$, and $\bar{y}_{ij} = \log \left[ 1 - p(j|i)\right]$, we can define

36 $$\begin{aligned} g(A,j)&= \sum _{A \in \mathcal {P}(S)} \exp \left[ \sum _{i \in A} y_{ij} + \sum _{i \in {A}^{\mathsf {c}}} \bar{y}_{ij} + b_A \right] , \end{aligned}$$

where $r(A,j) = g(A,j)$, with *g*(*A*, *j*) convex with respect to $y_{ij}$ and $\bar{y}_{ij}$ and invariant with respect to *p*(*j*|*i*) or *p*(*j*).

This gives the equivalent minimization problem:

37 $$\begin{aligned} \begin{array}{lclll} \displaystyle \underset{p(j|i)}{\text {minimize}} &{}{I(S;\widehat{S})} &{}&{}&{}\\ \text {subject to} &{} \mathbb {E}[g(A,j)] &{} \ge &{} r^* &{}\quad \forall j \in \widehat{S} \\ &{} p(j|i) &{} \ge &{} 0 &{} \quad \forall (i,j) \in (S,\widehat{S}) \\ &{} \sum _j p(j|i) &{} = &{} 1 &{} \quad \forall i \in S \\ &{} e^{y_{ij}} &{} \le &{} p(j|i) &{} \quad \forall (i,j) \in (S,\widehat{S})\\ &{} e^{\bar{y}_{ij}} &{} \le &{} 1-p(j|i) &{} \quad \forall (i,j) \in (S,\widehat{S}). \end{array} \end{aligned}$$

Setting aside non-negativity constraints on *p*(*j*|*i*) (these will be enforced by the form of the solution), we have the functional

38 $$\begin{aligned}&L\left[ p(j|i); p(j); y_{ij}, \bar{y}_{ij}\right] \nonumber \\&\quad = \sum _{i,j} p(j|i) \log \frac{p(j|i)}{p(j)} + \sum _i \lambda (i) \sum _j p(j|i) \end{aligned}$$

39 $$\begin{aligned}&\qquad - \beta \sum _{j, A \in \mathcal {P}(S)} \exp \left[ \sum _{i \in A} y_{ij} + \sum _{i \in {A}^{\mathsf {c}}} \bar{y}_{ij} + b_A \right] \end{aligned}$$

40 $$\begin{aligned}&\qquad + \sum _{i,j} \lambda (i,j) \left[ e^{y_{ij}} - p(j|i) \right] \end{aligned}$$

41 $$\begin{aligned}&\qquad + \sum _{i,j} \bar{\lambda }(i,j) \left[ e^{\bar{y}_{ij}} + p(j|i) \right] . \end{aligned}$$

We can then restate the original non-convex problem in terms of two convex minimizations and one quasiconvex minimization,

42 $$\begin{aligned} \min _{p(j|i)}\min _{p(j)}\min _{y_{ij},\bar{y}_{ij}} L\left[ p(j|i); p(j); y_{ij}, \bar{y}_{ij}\right] . \end{aligned}$$

Note that, similar to Tishby et al. (1999), the problem is not jointly convex and thus there is no guarantee of a unique global solution as in the rate-distortion case. Nevertheless, the marginal (quasi-)convexity admits an efficient iterative algorithm for identifying (locally) optimal solutions, similar to Tishby et al. (1999).

Taking the derivative of *L* with respect to *p*(*j*|*i*) and setting equal to zero, we arrive at

43 $$\begin{aligned} p(j|i)&= \frac{p(j)}{\mu (i)} \exp \left[ p(i)^{-1}\left[ \lambda (i,j) - \bar{\lambda }(i,j) \right] \right] , \end{aligned}$$

where $\mu (i)$ just normalizes the distribution over *j* for a given *i*. Taking the derivative of *L* with respect to $y_{ij}$ and setting equal to zero, we have

44 $$\begin{aligned} \lambda (i,j)&= \beta e^{-y_{ij}} \sum _{\{A \in \mathcal {P}(S)\,:\,i \in A\}} \exp \left[ \sum _{k \in A} y_{kj} + \sum _{k \in {A}^{\mathsf {c}}} \bar{y}_{kj} + b_A \right] . \end{aligned}$$

Doing the same for $\bar{y}_{ij}$ gives

45 $$\begin{aligned} \bar{\lambda }(i,j)&= \beta e^{-\bar{y}_{ij}} \sum _{\{A \in \mathcal {P}(S)\,:\,i \in {A}^{\mathsf {c}}\}} \exp \left[ \sum _{k \in A} y_{kj} + \sum _{i \in {A}^{\mathsf {c}}} \bar{y}_{kj} + b_A \right] . \end{aligned}$$

Subtracting the two equations, we have

46 $$\begin{aligned} \beta \Delta d(i,j)&= \lambda (i,j) - \bar{\lambda }(i,j), \end{aligned}$$

which is equivalent to the definition of $\Delta d(i,j)$ in the main text. Substituting into Eq. 43 produces

47 $$\begin{aligned} p(j|i)&= \frac{p(j)}{\mu (i)} \exp \left[ \frac{\beta }{p(i)} \Delta d(i,j)\right] . \end{aligned}$$

This gives the minimizing values of *L* with respect to *p*(*j*|*i*) for fixed *p*(*j*), $y_{ij}$, and $\bar{y}_{ij}$, as in Blahut (1972); Arimoto (1972); Tishby et al. (1999); Banerjee et al. (2005). The minimizing values of *L* with respect to *p*(*j*) are the same as in classical rate-distortion theory and are given by

48 $$\begin{aligned} p(j) = \sum _i p(j|i) p(i). \end{aligned}$$

The minimizing value of *L* with respect to $y_{ij}$ and $\bar{y}_{ij}$ under the constraints that $e^{y_{ij}} \le p(j|i)$, and $e^{\bar{y}_{ij}} \le \left[ 1 - p(j|i) \right]$, is simply

49 $$\begin{aligned} y_{ij}&= \log p(j|i), \end{aligned}$$

50 $$\begin{aligned} \bar{y}_{ij}&= \log \left[ 1 - p(j|i) \right] , \end{aligned}$$

since the monotonically decreasing 39 will achieve its minimum for the least negative values of $y_{ij}$ and $\bar{y}_{ij}$, which puts them up against their constraints.

### 1.3 Generalization

It is clear from the form of *g*(*A*, *j*) that the only requirement of the measured property, $b_A$, of any set, $A \in S$, is that it is nonnegative. Thus this same method may be employed for measures on sets other than redundancy, in the same way that rate-distortion theory treats generic measures of distortion. On the other hand, when the measured property offers certain kinds of additional structure, as in, e.g., the case of an average similarity (Slonim et al. 2005) measure, then other efficient solutions may be possible.

One variant to the sequential update of *p*(*j*|*i*) as listed in Fig. 8 is to modify every *p*(*j*|*i*) in parallel, which may be advantageous for some multiprocessor configurations. In practice, for convergence with simultaneous updating it appears to be important to introduce a slowdown factor, $\alpha$, to control the update of $p_t(j|i)$, i.e., using

51 $$\begin{aligned} p_t(j|i)&= \alpha \frac{p_t(j) e^{\beta \Delta d(i,j)}}{\sum _{j' \in \widehat{S}} p_t(j') e^{\beta \Delta d(i,j')}} + (1 - \alpha ) p_{t-1}(j|i), \end{aligned}$$

where *t* is the current iteration of the algorithm. The slowdown operates in a manner analogous to the learning rate in gradient descent optimization problems.

Like $\beta$, $\alpha$ does not require fine-tuning. It just needs to be small enough to allow for convergence, without being too small so as to allow the algorithm to converge in a reasonable number of iterations. While a more systematic investigation may be useful in identifying an efficient $\alpha$, we found that $\alpha = 0.1$ and $t = 200$ iterations was sufficient to ensure convergence for all the numerical results presented in the main text. In many cases a stable assignment is reached much earlier than after 200 iterations, and in general a stopping criteria based on the difference between assignments from one iteration to the next could be employed, though we did not do so here.

## 2 Simulation

The agent-based model used in this paper for generating schooling motion with known dependency structure is based on the three-zone-model introduced by Couzin et al. (2002). Each agent moves at a constant speed $s_0$ and responds to its conspecifics by changing its direction of motion. The interactions between individuals are governed by three basic social forces: long-range attraction, short-range repulsion, and intermediate-range alignment. However, there are two main differences from the original Couzin model: (1) the model is formulated in terms of stochastic differential equations with effective social forces (see Romanczuk et al. 2012; Romanczuk and Schimansky-Geier 2012); and (2) instead of discrete zones, we use overlapping social forces, whereby repulsion dominates at short distances $(r_{ij} < r_{\text {rep}})$, attraction dominates at long distances $r_{ij} < r_{\text {att}}$, and the alignment contribution overlaps with attraction and repulsion up to intermediate ranges $(r_{ij} < r_{\text {alg}})$, whereby $r_{\text {rep}}< r_{\text {alg}} < r_{\text {att}}$.

### 2.1 Model formulation

We simulate the movement of a group of *n* agents via a set of 2*n* (stochastic) differential equations. The agents move in a quadratic domain of size $L\times L$ with periodic boundary conditions. The dynamics of each agent (in 2d) are described by the following equations of motion $(i = 1,\dots , n)$:

52 $$\begin{aligned} \frac{d \mathbf {r}_i}{dt}= \mathbf {v}_i(t), \qquad \text {with}\quad \mathbf {v}_i(t) = {s_0\cos (\varphi _i(t)) \atopwithdelims ()s_0\sin (\varphi _i(t)) }, \end{aligned}$$

53 $$\begin{aligned} \frac{d \varphi _i}{dt}= & {} \frac{1}{s_0}\left( F_{i,\varphi } + \eta _{i,\varphi } \right) . \end{aligned}$$

Here $\mathbf {r}_i$, and $\mathbf {v}_i$ are the Cartesian position and velocity vectors of each agent, with $s_0$ being the (constant) speed of agent *i*. Furthermore, $\eta _{i,\varphi }$ are Gaussian white noise terms accounting for randomness in the turning motion of individuals, and $\mathbf {F}_{i,\varphi }$ are the projections of the total social forces inducing turning behavior, where

54 $$\begin{aligned} F_{i,\varphi }=\mathbf {F}_i \cdot \mathbf {u}_{\varphi ,i} = \mathbf {F}_i {- s_0\sin \varphi _i \atopwithdelims ()s_0\cos \varphi _i }. \end{aligned}$$

The total effective social force is a sum of three components, $\mathbf {F}_i=\mathbf {F}_{i,\text {rep}}+\mathbf {F}_{i,\text {alg}}+\mathbf {F}_{i,\text {att}}$,

55 $$\begin{aligned} \begin{array}{rcl} \text {Attraction} &{} &{} \displaystyle \mathbf {F}_{i,\text {rep}}=\sum _{j \in \text {Neigh}} +\mu _{\text {att}}S_{\text {att}}({r}_{ji}) \mathbf{\hat{r}}_{ji}, \\ \text {Repulsion} &{} &{} \displaystyle \mathbf {F}_{i,\text {rep}}=\sum _{j \in \text {Neigh}} -\mu _{\text {rep}}S_{\text {rep}}({r}_{ji}) \mathbf{\hat{r}}_{ji}, \\ \text {Alignment} &{} &{} \displaystyle \mathbf {F}_{i,\text {alg}}=\sum _{j \in \text {Neigh}} \mu _{\text {alg}}S_{\text {alg}}({r}_{ji}) (\mathbf {v}_j-\mathbf {v}_i), \end{array} \end{aligned}$$

with $\mathbf{\hat{r}} = \mathbf {r}/|\mathbf{r}|$. The strength of the different interactions is set by a constant $\mu _X$ and a sigmoid function of distance, which goes from 1 to 0, with the transition point at $r_{X}$ and steepness $a_{X}$:

$$\begin{aligned} S_X(r)=\frac{1}{2}\left( \tanh (-a(r-r_{X})+1\right) \end{aligned}$$

(Fig. 9).

The stochastic differential equations for the direction of motion of individual agents are solved by a simple Euler-Maruyama method:

56 $$\begin{aligned} \varphi (t+1)= & {} \varphi (t) + \frac{1}{s_0}\left( F_{i,\varphi }(t)\Delta t + \sqrt{2 D_\varphi \Delta t} \text { GRN(t)}\right) , \end{aligned}$$

57 $$\begin{aligned} \mathbf {r}(t+1)= & \mathbf {r}(t) + {s_0\cos (\varphi _i(t)) \atopwithdelims ()s_0\sin (\varphi _i(t)) }\Delta t. \end{aligned}$$

### 2.2 Numerical experiments

We simulated independent groups of three different sizes, $n = 5$, 10, and 15, wherein it was possible for each agent to interact with the distance dependent effective forces with all other agents within the group. The initial conditions were always a random distribution of agents in the simulation domain with random initial direction of motion. In order to ensure formation of a single cohesive group we set the attraction range to be larger then the domain size $r_{\text {att}}>L$. In all simulation runs considered here, we obtained for the used parameters (see Tab. 1) a single polarized group after a transient time of $t < 400$. Thus for our analyses we used only data for $t > 400$.

**Table 1:** Parameter values used in the simulations

Parameter	Symbol	Value
Domain size	*L*	100
Repulsion range	$r_{\text {rep}}$	1.0
Attraction range	$r_{\text {att}}$	100.0
Alignment range	$r_{\text {alg}}$	5.0
Repulsion strength	$\mu _{\text {rep}}$	2.0
Attraction strength	$\mu _{\text {att}}$	0.3
Alignment strength	$\mu _{\text {alg}}$	0.8
Steepness of interaction function	*a*	10
Speed of individuals	$s_0$	1.0

## Footnotes

1 As noted by Watanabe (1960), its significance as a potential measure of organization stretches back still further, to at least Rothstein (1952).

2 Meaning that they share the same outcome.

3 If $m > n$ then some components will necessarily be empty.

4 The use of *i* and *j* as elements of *S* and $\widehat{S}$, respectively, will follow this convention in the rest of the paper.

5 Unless stated otherwise, the complement of a set is taken with respect to *S*, i.e., ${A}^{\mathsf {c}} = \{k \in S : k \not \in A \}$.

6 Using the simultaneous updating variant of the algorithm, see Appendix 1.

7 This figure corrects an earlier preprint of this manuscript. The early draft contained a numerical error in the computation of the log determinant used in the average redundancy bounds, which artificially reduced the estimated average redundancy for large systems with small numbers of components.

8 In both noise conditions all three non-interacting groups were split into separate components. In the low noise condition, the group of 20 was further subdivided into two components.
